# Cloud Services for Patient Cohort Identification Using the Informatics for Integrating Biology and the Bedside Platform

**DOI:** 10.1155/2020/2851713

**Published:** 2020-07-07

**Authors:** Kavishwar B. Wagholikar, Shreekanth V. Joshi, Vishal V. Pai Vernekar, Yuri Ostrovsky, Somnath D. Desai, Pooja B. Magdum, Sachin B. Wakle, Sheetal Jain, Akshay Zagade, Rahul Patel, Shawn N. Murphy

**Affiliations:** ^1^Harvard Medical School, Boston, MA, USA; ^2^Massachusetts General Hospital, Boston, MA, USA; ^3^Partners Healthcare, Boston, MA, USA; ^4^Persistent Systems, Pune, India

## Abstract

Despite the widespread use of the “Informatics for Integrating Biology and the Bedside” (i2b2) platform, there are substantial challenges for loading electronic health records (EHR) into i2b2 and for querying i2b2. We have previously presented a simplified framework for semantic abstraction of EHR records into i2b2. Building on our previous work, we have created a proof-of-concept implementation of cloud services on an i2b2 data store for cohort identification. Specifically, we have implemented a graphical user interface (GUI) that declares the key components for data import, transformation, and query of EHR data. The GUI integrates with Azure cloud services to create data pipelines for importing EHR data into i2b2, creation of derived facts, and querying for generating Sankey-like flow diagrams that characterize the patient cohorts. We have evaluated the implementation using the real-world MIMIC-III dataset. We discuss the key features of this implementation and direction for future work, which will advance the efforts of the research community for patient cohort identification.

## 1. Introduction

I2b2 has been widely deployed to enable researchers to identify patient cohorts for clinical studies [1, 2]. Over 200 institutions have deployed i2b2 worldwide, and the deployments at several institutions in the United States are connected into networks for federated querying [3, 4]. However, despite the widespread use of the i2b2 platform, there remain substantial challenges for importing EHR data into i2b2 and for querying the data in i2b2.

First, there currently exist no good practice guidelines or tooling that information technology (IT) teams can use to import EHR data into i2b2, and the IT team faces a steep learning curve to understand the i2b2 web services and database schema to load data into i2b2 [5, 6]. Due to lack of tooling, IT teams resort to ad hoc methods to import the data. They develop data import pipelines and also perform the “Devops” tasks to create and manage the computational environment for running the pipelines. Consequently, considerable manual effort is expended on data import.

Secondly, although the i2b2 web client interface allows development of complex queries, it is challenging for most users to construct queries for complex patient cohorts. Moreover, there is no functionality to visualize the query results or understand the effect of different filters to gain a better perspective of the patient cohort. For instance, it is nontrivial to query patients with a last laboratory value (e.g., last serum glucose > 176 mg/dl). Several such criteria need to be combined to represent the eligibility criteria for most clinical studies, and it is tedious to examine the effect of individual criteria on the patient count, using the i2b2 web client.

To address the above shortcomings, building on our previous work, we have implemented a proof-of-concept system using cloud-based services to augment patient cohort identification on the i2b2 data store. In our previous work, we had described a framework involving four facets: (1) transforming EHR into a simplified schema for import into i2b2; (2) use of standard vocabularies to create a concept catalogue; (3) mapping local codes to those from standard vocabularies; and (4) definition of clinically meaningful “derived concepts” that involve computation on imported i2b2 facts, resulting in generation of “derived facts” [7]. Together, these four facets facilitate data import and simplify querying. However, our previous implementation lacked a GUI and a mechanism to compute derived facts.

Building on the previous work, we have now implemented a scalable system that links a graphical user interface (GUI) to Microsoft Azure cloud services. The GUI enables data analysts to define processes for data import and transformation, and the back-end cloud services then execute the defined processes. Furthermore, the GUI enables clinical users to construct queries using Sankey-like flow diagrams, which are rendered by the cloud services. We have evaluated this system using the real-world MIMIC-III dataset.

The main contribution of this paper is to describe the functionality needed to use cloud services to load and transform EHR data into an i2b2 patient store. Another contribution is the graphical search interface that serves an alternative to the i2b2 search web interface. We anticipate that our system will minimize the ad hoc programming effort to load and transform EHR data in i2b2 and that the flow diagram-based GUI will facilitate querying for patient cohort. Overall, the design of our system will inform other implementations and tools for patient cohort identification.

## 2. Methods

We define two types of users or roles for the system: (1) knowledge engineer or data analyst and (2) primary/clinical user. The knowledge engineer sets up the system by loading data and transforming it into a query-friendly form, and the primary user queries the system to generate patient cohorts.

**Figure 1:**
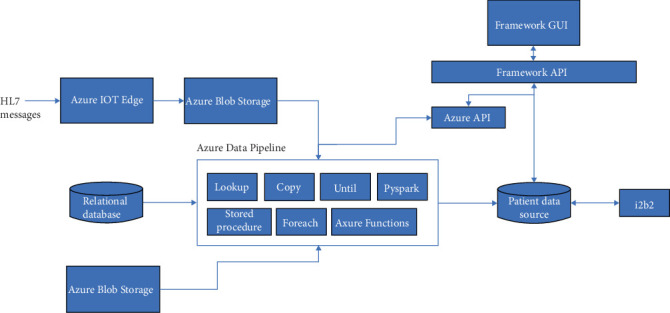
High-level technical block diagram.

We implemented a GUI for knowledge engineers to set up an i2b2 data store and for primary users to easily create and execute complex queries and to visualize their results. The GUIs are connected at the back end to the Azure Data Factory application programming interface (API).  Figure 1 below provides the architectural overview of the system. Framework GUI is an Angular-based web application for authoring. It uses the Framework API which is a Java-based REST API that wraps around Azure Data Pipeline REST API. The Framework API can be used to create metadata and to configure Azure Data Pipelines. The resulting Azure Data Pipelines can ingest data from data sources such as relational database, file system, and streaming HL7 messages and can transform the ingested data to create derived data using either Azure Functions, stored procedures, lookups, or a Pyspark script. Patient data store uses the i2b2 schema to store observation fact data. Videos demonstrating the features of the system are available at the following link.

The web-based GUI enables a knowledge engineer to define the logical components of a data construct we refer to as “Logic” (described below). When the engineer saves a Logic, the Azure Data Pipelines API is called in the back end to create an Azure-managed data pipeline. The pipeline can be immediately executed or scheduled to run at a particular time or run when triggered by an event. When a data pipeline is triggered, an instance of execution of the pipeline is created. An executed data pipeline reads data from a source, performs the appropriate transformation, stores it in the i2b2 observation_fact table, and outputs logs.

### 2.1. Knowledge-Modeling GUI

We have implemented a construct called Logic, which can be one of 4 types: (1) *import-logic*: to import EHR data; (2) *concept-hierarchy logic*: for creating concepts, along with their standard codes in the i2b2 concept hierarchy; (3) code *map logic*: to map the imported local data to a standard coding system; and (4) *derived-concept logic*: to transform the imported data into clinical meaningful “derived concepts.” Examples of the four types of “Logics” are as follows:

1. The Labs-Import *import-logic* sets up an Azure job to query the staging repository for most of the laboratory data, which is inserted into the i2b2 database including the fact, patient, provider, and encounter tables
2. The Lab-Hierarchy is a *concept-hierarchy logic* that imports the LOINC coding system from UMLS to create a concept hierarchy. For instance, it creates a concept “Serum Glucose”: glucose [mass/volume] within the i2b2 ontology under the heading of “Serum or Plasma” with LOINC code 2345-7 as a leaf node with path Labs/…/Serum Glucose
3. The Serum-Glucose-Mapping is a *concept-map logic* that maps the local codes for glucose to the LOINC code 2345-7 and links the fact table to the standard concept definition. It does so by extending the i2b2 hierarchy by adding the local codes as children of the standard codes
4. “Last-Serum-Glucose” is a *derived-concept logic* to create a derived concept of the same name, where the facts for serum glucose for each patient are sorted by date, and the last one is copied as last serum glucose

### 2.2. Logic Modality

The logics can be defined in one of four modalities: Structured Query Language (SQL), a file following the i2b2-cdi format, Python code (Pyspark), or Azure Functions. Typically, SQL can be used to implement the import-logics for data in a database source system (e.g., MIMIC-III). SQL can also be used to compute derived facts (e.g., last serum glucose > 176 mg/dl). Loading data from a file compliant with the i2b2-cdi format will implement both import-logic and local-concept-map logic using metadata within the file. We recommend the use of Pyspark-based algorithms for computing complex derived concepts like for risk scores and machine learning models [8]. Simple computations that complete within a few minutes can be implemented with Azure Functions that can be written in a variety of programming languages (e.g., Java, C#, and TypeScript).

**Figure 2:**
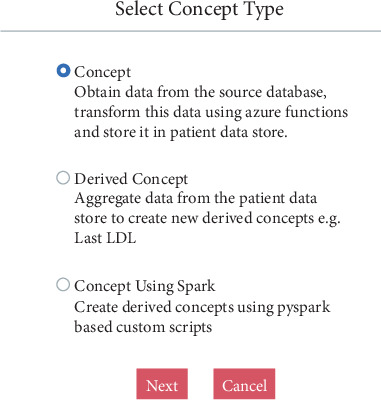
Creating concepts and derived concepts.

Knowledge engineering GUI supports creation of concept and derived concepts as show in Figure 2 below.

**Figure 3:**
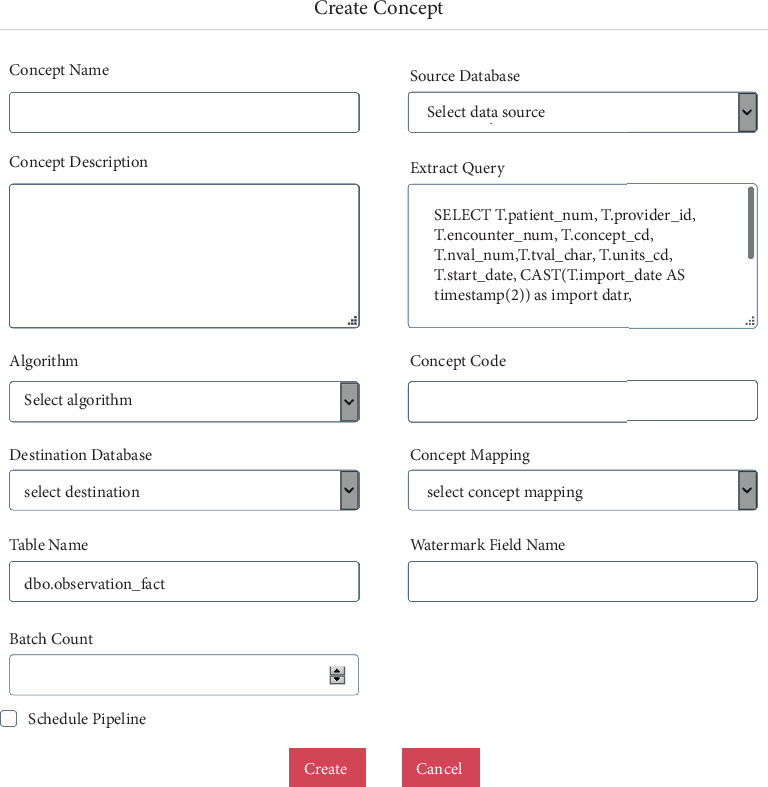
Creating a concept.

Knowledge engineering GUI supports creation of a concept as shown in Figure 3. Creation of a concept, e.g., LDL, sets up an Azure Data Pipeline to ingest LDL data from source system into the system. The below UI shows an example of configuring the source system and query to extract data from the source system incrementally, apply Azure Function and code mapping to transform data, and store in the patient data store.

Most of the fields are self-explanatory. Below are the ones that need description:

- Extract query—query that will be used to extract data from the source system
- Algorithm—Azure function that can be used to transform the incoming data
- Concept Mapping—local to standard or standard to local code mapping
- Watermark Field Name—incremental loading
- Batch count—batch size that is used to read the data from the source system

**Figure 4:**
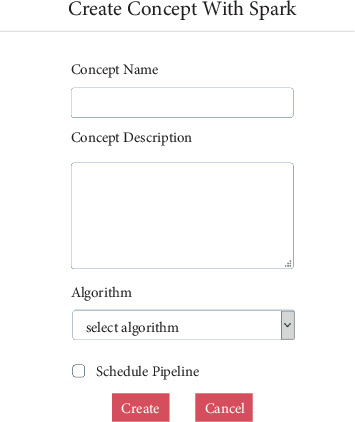
Creating a derived concept using Pyspark.

Knowledge engineering GUI supports creation of a derived concept as show in Figure 4. Most of the fields are self explanatory. Below are the ones that need description:

- Algorithm—Pyspark script that contains the algorithm

Once a concept/derived concept is created, it is reusable and can be used to build various cohorts using the query construction GUI.

### 2.3. Query Construction GUI

**Figure 5:**
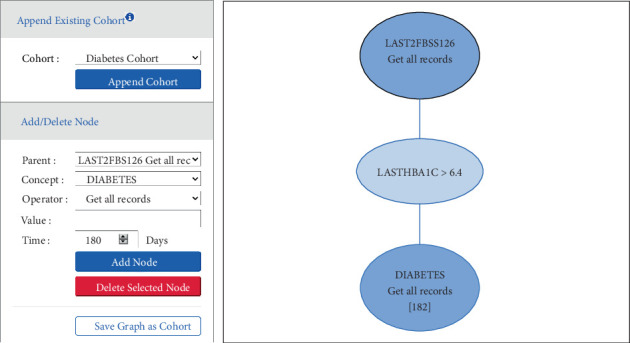
Graphical user interface for cohort querying. The flow diagram shows each node in the selection criteria.

Researchers can add the concepts (standard and derived) defined by the knowledge engineer to form filters/nodes that go into a flow diagram, as shown in Figure 5.

Note that sibling node represent “OR” condition and children nodes represent “AND” in the cohort querying UI. Within each node, several operators are supported, e.g., >, <, and ≥, within a time window. Any complex query (e.g., algorithm, temporal, and exclusion) requires to be first decomposed into constituent criteria, and then, the concepts representing each criteria are selected in the GUI to build the query.

### 2.4. Connection to Azure API

GUI uses the Azure Data Factory REST API to create Azure data jobs. Azure Data Factory REST API is a cloud data integration service to compose data storage, movement, and processing services into automated data pipelines [9].

### 2.5. Azure Data Jobs

Azure data jobs are managed Azure Data Pipelines that contain various activities like lookup activity, copy data activity, and stored procedure activity that are chained together. Azure Data Pipelines can be manually triggered or can automatically run at specified frequency or trigged based on an event. Azure Data Pipelines also provide the ability to monitor the jobs and to view logs.

### 2.6. Evaluation

To evaluate the performance of the system, we imported a subset of the MIMIC-III dataset [10], using the GUI to define Logics for importing the data, creating derived facts, and for querying for diabetes patients.

We created an Azure SQL database with a Max storage space of 250 GB, on a standard S2 (50 data throughput unit) pricing tier in the West US location. The source database used for the evaluation was a Postgres database running on CentOS7 on a machine with 8 GB RAM and 2 CPUs on an Azure VM.

MIMIC-III (Medical Information Mart for Intensive Care III) dataset was loaded into the Postgres databases from the SQL dump files following the documented installation instructions [10]. MIMIC-III is a large, freely available database comprising deidentified health-related data associated with over 40,000 patients who admitted to critical care units of the Beth Israel Deaconess Medical Center between 2001 and 2012. The database includes information such as demographics, vital sign measurements made at the bedside, laboratory test results, procedures, medications, caregiver notes, imaging reports, and mortality (both in and out of hospital). We imported the diagnosis and laboratory records from this dataset. The diagnoses were encoded using the International Classification of Diseases version 9 (ICD-9) coding system, and the laboratory records were encoded using “Logical Observation Identifiers Names and Codes” (LOINC) coding system.

## 3. Results

For querying for a cohort of diabetic patients from the MIMIC-III dataset, we created Logics for importing relevant data and for creating the required derived facts, as summarized in Table 1. For example, the first row of table denotes that SQL query for importing diagnosis is named Diagnosis-import, and catalogued with hierarchical path Import/Diagnosis-import. Execution of the system resulted in the import of all of the 651,047 diagnoses and 27,854,055 laboratory records from the MIMIC-III dataset.  Table 2 shows the criteria for the diabetes cohort.

**Figure 6:**
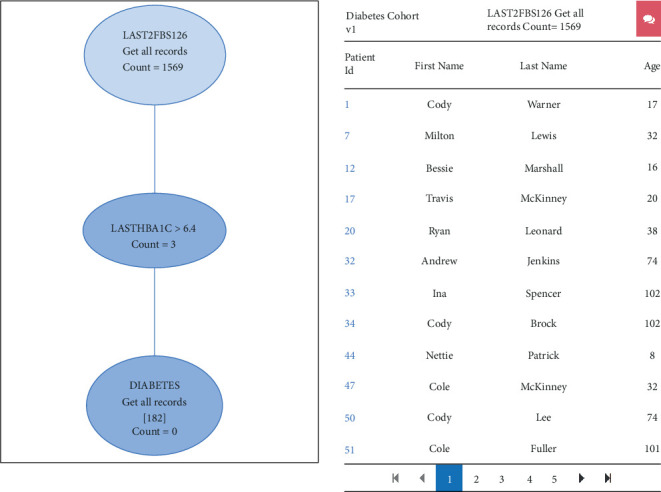
Flow diagram summarizes the result of the query, where each node shows the patient count resulting from filters in the current node and parent.

The flow diagram resulting from the query for the diabetes cohort is shown in Figure 6 below. It shows the criteria as nodes along with the count of patients that matched the criteria. Selection of a node expands the view in the right pane which shows the details of the patients that matched the criteria. Clicking on a particular patient row gives the entire patient chart for the selected patient.

## 4. Discussion

We evaluated our system for querying the MIMIC-III dataset for diabetes patients. The results demonstrate that the “Logic construct” is useful to capture the knowledge required to import data from an external repository and to transform it into a query-friendly form and that the GUI successfully provides the functionality to visualize the results of a cohort query [11]. The four Logic types cover the process of moving the data into the i2b2 repository, while mapping it to standard vocabulary and transforming it into clinically meaningful intermediate “derived” concepts that are semantically closer to the target query.

### 4.1. Data Transformations

Our system presents an implementation of “derived concepts” that were introduced in our previous work. The primary advantage of derived concepts, in the context of querying, is that they allow a complex query to be broken down into modular components. Without the “derived concepts,” it would be very difficult (if not impossible, given the limitations of the i2b2 query tool) to execute the query for a diabetes cohort described in this manuscript. For instance, is it nontrivial to define the criteria of “Last HbA1c” using the temporal querying capability of the i2b2 web client. Moreover, each of the criteria would have to be defined as queries in themselves and then joined into a compound query by dragging the query components into the web client query windows. Hence, by availing assistance from the knowledge engineer who can create the required derived concepts, the clinical user can easily build complex queries that were not possible before.

However, the creation of derived concepts requires them to be defined in one of the implementation modalities, which is beyond the expected knowhow of a typical “clinical” user desirous of querying the i2b2 instance. We anticipate the derived concept queries to be created by the knowledge engineer and then precomputed and made available for querying by the primary users. Consequently, a disadvantage of this approach is that the advanced querying capabilities of primary users are limited to the availability of a set of predefined derived concepts created by the knowledge engineer. This can, however, also be viewed as an advantage, as it encourages standardization and correctness of the underlying concepts. Currently, only the knowledge engineer can define the derived concepts, although we anticipate that this will translate to a privileged role that can be assigned to primary users who have the technical knowhow to create derived concepts.

### 4.2. Reusability

As mentioned above, the derived concepts created by the knowledge engineer are available to the primary user to build queries from. The derived concepts created for a particular use case or project may likely be useful for other projects.

### 4.3. Clinical Inference Layer

Derived concepts essentially embed medical knowledge that yields an inference layer in the form of inferred/derived facts around the raw patient data. For instance, the knowledge that last HbA1C > 6.4% is a clinically significant fact and that it is indicative of diabetes is available for the primary user in the form of derived concepts with canonical paths: /Derived/Labs/Blood/Chemistry/HbA1c/LastHbA1c>percent and /Derived/Diagnosis/Diabetes/lastHbA1c>6.4%, respectively.

### 4.4. Manage Complexity

The extract, transform, and load (ETL) process for creating an i2b2 repository is inherently complex as multiple sources and transformations are involved. Our system decomposes the entire process into named components that are organized in a “Logic hierarchy” which facilitates code organization and debugging.

As shown in Table 1, the path of each “logic” component is used to create a hierarchical catalogue that helps find the component.

### 4.5. Automated Orchestration

A key feature of our system is that the GUI enables the authoring of the components needed to move and transform the data. The web services orchestrate the creation, execution, and destruction of processes to execute the data movement and transformations. In the absence of the Azure data pipe services, the Health IT team would need to write the programs to create daemons for accomplishing the above tasks and would also need to perform the “devops” tasks to create and manage the computational environment for running the daemons [6, 12]. Use of cloud services minimizes the need for writing programs for orchestration of the data processing and management of the compute environment, which significantly reduces manual programming effort.

### 4.6. Cohort Visualization

Our evaluation also demonstrates (see video at link <https://www.youtube.com/watch?v=4mCZRAFI_9U>) that the query GUI successfully provides the drag and drop functionality to create a flow diagram that shows the effect of query criteria, as attritions in patient counts. For instance, the flow diagram in the diabetes example query that we generated shows that the query by diagnostic codes yields a large number of patients, as it has high recall. Querying by stringent criteria like using the last HbA1C > 9.4%, yields a smaller set that is expected to have high positive predictive value. Adding a criteria like “last two serum glucose > 120” further improves the precision at the cost of recall. The investigator can decide on the precision of the query and choose the suitable criteria using the flow diagram as a guide. While querying by the individual criteria is possible using the i2b2 web client, our GUI provides rapid insight about the query performance by generating the flow diagram.

### 4.7. Cloud Service Provider

Although we have utilized the Azure cloud service to develop our system, we anticipate that our system can be adapted or ported to data processing services on the Google and Amazon cloud using the data pipeline constructs in the respective cloud [13].

### 4.8. Limitations

A limitation of our evaluation is that we did not evaluate the “local-concept-map” Logic, as the MIMIC-III dataset already has standard codes for diagnosis and laboratory result, i.e., ICD and LOINC, respectively. Second, we limited our derived concepts to simple rules although machine learning models can be modeled using our current implementation mechanism [14]: our system supports machine learning via Pyspark-based algorithms that can run within a data pipeline. Our systems provide the mechanism to execute a programming script that results into a derived fact for each patient. This functionality can be used to deploy machine learning models. For instance, models to compute risk score of a particular disease can be deployed.

Third, we have currently not addressed the problem of circular dependency that can arise when two derived concepts are defined as referencing each other. Fourth, we expect that the i2b2 repository generated by our system will seamlessly integrate with the i2b2 wildfly services and will be queryable using the web client; but we have not validated this currently.

Finally, we have limited our evaluation to test the functional aspects of the system. A rigorous usability evaluation is required with real-world use cases to determine if the system truly can reduce the manual effort to load and transform EHR data into i2b2 and whether the flowchart query interface can augment the search capabilities already provided by the i2b2 web client.

## 5. Concluding Remarks and Future Work

We have implemented a system that allows the knowledge engineer to define the components needed for importing EHR data and for transforming it into a form that facilitates querying. As the defined components are automatically converted into processes managed by the cloud service, our system significantly reduces the need for manual programming and for managing the compute environment. The systems' graphical interface facilitates querying for patient cohorts, by providing a quantitative visualization of the query results as a flow diagram. The design of the system will inform other implementations and tools for patient cohort identification. Our future work includes evaluation of usability of the system in a real-world setting and benchmarking the scalability of the system on larger datasets.

## Figures and Tables

**Table 1:** Logic definitions.

Logic type	Name	Logic path	Modality of implementation
Import	Diagnosis-import	Import/Diagnosis-import	SQL query
Import	Labs-import	Import/Labs-import	SQL query
Concept hierarchy	Diagnosis hierarchy	Concept_Hierarchy/Diagnosis-hierarchy	Tab separated file
Concept hierarchy	Labs hierarchy	Concept_Hierarchy/Labs-hierarchy	Tab separated file
Derived concept: Boolean	Diagnosis-diabetes	Derived_Concept/Diagnosis-Diabetes	SQL query
Derived concept: numeric	Last HbA1c	Labs/BloodTest/Chemistry/HbA1c/Last HbA1c	SQL query
Derived concept: numeric	Serum glucose	Labs/BloodTest/Chemistry/Glucose/Last-Glucose	SQL query
Derived concept: Boolean	Last two consecutive serum glucose > 126	Labs/BloodTest/chemistry/glucose/last-twoSerumGlucose/LastTwoSerumGlucose_ > _126	SQL query

**Table 2:** Diabetes cohort criteria.

Rationale for filter	Description for filter
Based on ICD9 codes	Diagnoses code of diabetes
Based on serum glucose	Last two consecutive serum glucose > 126
Based on HbA1c	Last HbA1c > 6.4

## Data Availability

The data used in this paper is available on request at https://mimic.physionet.org/.
